# Evaluation of clonal origin of malignant mesothelioma

**DOI:** 10.1186/s12967-014-0301-3

**Published:** 2014-12-04

**Authors:** Sabahattin Comertpay, Sandra Pastorino, Mika Tanji, Rosanna Mezzapelle, Oriana Strianese, Andrea Napolitano, Francine Baumann, Tracey Weigel, Joseph Friedberg, Paul Sugarbaker, Thomas Krausz, Ena Wang, Amy Powers, Giovanni Gaudino, Shreya Kanodia, Harvey I Pass, Barbara L Parsons, Haining Yang, Michele Carbone

**Affiliations:** University of Hawaii Cancer Center, University of Hawaii at Manoa, Honolulu, HI USA; Department of Health Sciences, Università del Piemonte Orientale “Amedeo Avogadro”, Novara, Italy; Department of Melocular Biology and Bioengineering, University of Hawaii at Manoa, Honolulu, HI USA; Cardiothoracic Surgery, Maine Medical Center, Portland, ME USA; Penn Presbyterian Medical Center, Philadelphia, PA USA; Program in Peritoneal Surface Malignancy, MedStar Washington Hospital Center, Washington, DC USA; Department of Pathology, University of Chicago, Chicago, IL USA; Sidra Medical and Research Centre, Doha, Qatar; Department of Biomedical Sciences and Samuel Oschin Comprehensive Cancer Institute, Cedars-Sinai Medical Center, Los Angeles, CA USA; Department of Cardiothoracic Surgery, New York University, New York, NY USA; Division of Genetics and Molecular Toxicology, National Center for Toxicological Research, US FDA, Jefferson, AR USA; Department of Pathology, John A. Burns School of Medicine, University of Hawaii, Honolulu, HI USA

**Keywords:** Malignant mesothelioma, Clonal origin, HUMARA assay, Carcinogenesis, Polyclonal tumors

## Abstract

**Background:**

The hypothesis that most cancers are of monoclonal origin is often accepted as a fact in the scientific community. This dogma arose decades ago, primarily from the study of hematopoietic malignancies and sarcomas, which originate as monoclonal tumors. The possible clonal origin of malignant mesothelioma (MM) has not been investigated. Asbestos inhalation induces a chronic inflammatory response at sites of fiber deposition that may lead to malignant transformation after 30-50 years latency. As many mesothelial cells are simultaneously exposed to asbestos fibers and to asbestos-induced inflammation, it may be possible that more than one cell undergoes malignant transformation during the process that gives rise to MM, and result in a polyclonal malignancy.

**Methods and results:**

To investigate the clonality patterns of MM, we used the HUMARA (Human Androgen Receptor) assay to examine 16 biopsies from 14 women MM patients. Out of 16 samples, one was non-informative due to skewed Lyonization in its normal adjacent tissue. Fourteen out of the 15 informative samples revealed two electrophoretically distinct methylated HUMARA alleles, the Corrected Allele Ratio (CR) calculated on the allele peak areas indicating polyclonal origin MM.

**Conclusions:**

Our results show that MM originate as polyclonal tumors and suggest that the carcinogenic “field effect” of mineral fibers leads to several premalignant clones that give rise to these polyclonal malignancies.

**Electronic supplementary material:**

The online version of this article (doi:10.1186/s12967-014-0301-3) contains supplementary material, which is available to authorized users.

## Introduction

Malignant mesothelioma (MM) is an aggressive cancer arising from the transformation of the mesothelial lining of the pleura, peritoneum and pericardium. It is a lethal cancer affecting approximately 3,200 individuals each year in the US, most of them die within 1 year from diagnosis [1]. MM incidence has remained stable in the US since 2003, but it continues to increase worldwide, due to exposure to asbestos fibers, which are widely used for industrial purposes [1]. Occupational exposure accounts for a male to female MM incidence ratio of 6-8 to 1, as males are much more often involved in the asbestos industrial trades [2]. Asbestos, erionite and other mineral fibers are also naturally present in developing rural areas where they pose a major risk factor for MM [3,4]. Asbestos inhalation induces a chronic inflammatory response at sites of fibers deposition that may lead to malignant mesothelial cell transformation after a latency of 30- to 50-years [5]. In addition to the environmental and inflammatory components of MM etiology, we have recently discovered that germline mutations of the *BAP1* gene cause a novel cancer syndrome characterized by a very high incidence of MM, and other malignancies [6].

MMs show different histology. There are 3 main subtypes: about 50% of MM show an epithelioid morphology and they look like carcinomas, 10% have a spindle cell morphology similar to sarcomas, and about 35% are biphasic, composed of both epithelioid and spindle cells in different proportions. In addition there are less common histological variants [7]. Diagnosis is further complicated by the presence of intra-tumoral pleomorphisms and phenotypic heterogeneity, raising the question of whether MM results from genetic and epigenetic alterations, which drive clonal tumor evolution into these different morphologies, or it arises from different subsets of mesothelial cells that interact or cooperate to drive malignant progression.

Multistep carcinogenesis is the currently accepted hypothesis to explain genetic diversity in tumors [8-10]. This hypothesis is based on the notion that somatic mutations are rare events that are unlikely to co-occur in different single normal cells, and that the mutations leading to the genetic diversification of the tumor architecture occur during the process of clonal expansion and selection. A cancer is considered monoclonal when all cells within the tumor can be traced back to a single progenitor/initiator cell. Instead, a polyclonal malignancy derives from the concomitant transformation of two or more different ancestor cells. Determining the clonal status of a cancer can be quite challenging, because of the inherent plasticity of the cancer genome that can acquire many somatic mutations during malignant cell growth. Analysis of the X chromosome inactivation pattern in female cancer biopsies, by measuring the methylation status of the polymorphic human androgen receptor (*HUMARA*) locus, is considered the most accurate method to assess clonality [11]. During early female embryogenesis, one of the two X chromosomes is randomly inactivated and the pattern of X-chromosome inactivation is stably transmitted from parent cell to the progeny (Lyonization) [12]. Therefore, the presence of the same inactivated X chromosome in all cancer cells has been interpreted as an indication of monoclonality. Although the current dogma is that cancers originate as monoclonal growths, some studies suggest that some cancers may arise as polyclonal [13,14].

To the best of our knowledge, the clonal origin of MM has never been investigated. However MMs are “assumed” to be monoclonal as most tumors are usually assumed to be. Here we tested the hypothesis that MMs are monoclonal using the HUMARA assay on 16 MM biopsies from 14 female patients. We found that human MMs are polyclonal in origin.

## Methods

### Clinical specimens and study approval

Human MM biopsies were collected at the following institutions: Department of Cardiothoracic Surgery, New York University, New York, NY; MedStar Washington Hospital Center, Washington, DC; University of Wisconsin School of Medicine and Public Health Department of Surgery, Madison, WI, and at the Department of Surgery, Penn Presbyterian Medical Center, Philadelphia, PA.

All tissue collections were approved by the Institutional Review Boards. Written and informed consent was obtained from all patients included in the study according to the guidelines set forth by the Institutional Review Board and in agreement with the Helsinki Declaration of 1975, as revised in 1983. Specimens were de-identified prior to analysis. Tissues collected during surgical tumor resection were immediately frozen and processed for laser microdissection, DNA extraction and immunohistochemistry. Clinical features are included in Table 1. Laser microdissection and all following experimental procedures were conducted at the University of Hawai‘i Cancer Center.

**Table 1:** **Clinical features and clonality pattern in 16 biopsies from 14 female MM patients**

**Sample ID**	**Age**	**Histology**	**Staging**	**Clonality**
**6**	ND	Biphasic	NA	Polyclonal
**61**	82	Epithelioid	III	Polyclonal
**93**	64	Epithelioid	III	Polyclonal
**207A**	74	Epithelioid	III	Polyclonal
**207B**				Polyclonal
**273**	66	Biphasic	III	Polyclonal
**524A**	72	Epithelioid	III	Polyclonal
**524B**				Monoclonal
**851**	58	Biphasic	III	Polyclonal
**1250**	65	Epithelioid	III	Polyclonal
**1359**	56	Epithelioid	III	Polyclonal
**1419**	25	Biphasic	III	Polyclonal
**L-III-18**	64	Epithelioid	III	Non-informative
**R693**	63	Epithelioid	III	Polyclonal
**R908**	69	Epithelioid	I	Polyclonal
**W-III-6**	66	Epithelioid	II	Polyclonal

### *HUMARA* assay

**Figure 1:**
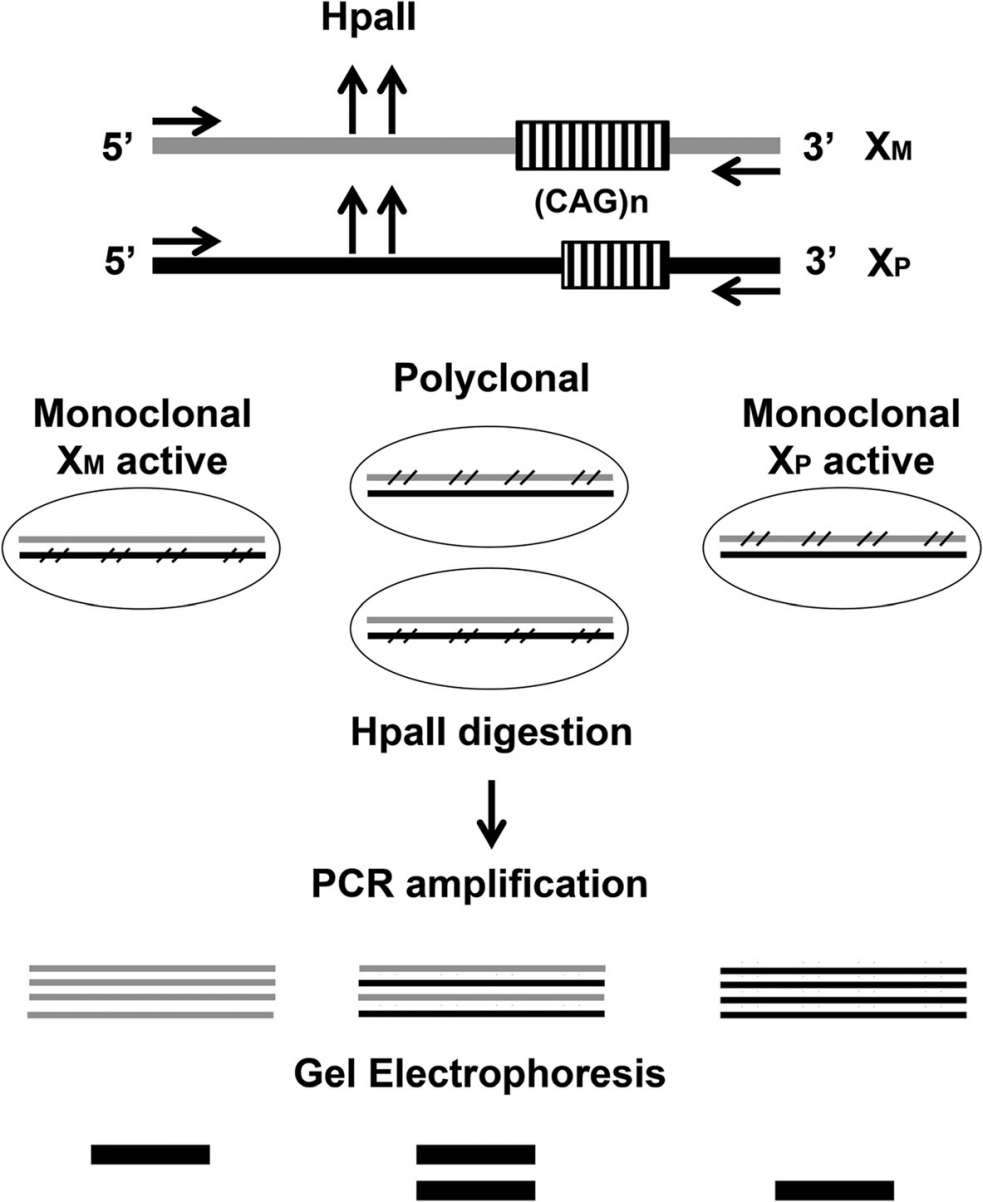
**Schematic diagram of the** ***HUMARA*** **assay.** Maternal and paternal X chromosomes carry different numbers of CAG repeats at the Humara locus. HpaII methylation sensitive sites are located at the polyorphic CAG region. During embriogenesis, random X chromosome inactivation occurs in female individuals, resulting in methylation of either the paternal or maternal X chromosome in different cells. Therefore, a monoclonal cell population, derived from the division of a single ancestor cell, shares the same inactivated X chromosome, whereas a polyclonal population, derived from more than one ancestor cell, may contain cells with inactive maternal and paternal X chromosomes. HpaII digestion removes the unmethylated alleles, allowing amplification of the methylated *HUMARA* locus. Electrophoresis of the PCR products will indicate monoclonal or polyclonal cell populations, as a single band or two bands of different size, respectively. HpaII: denotes the methylation sensitive endonuclease sites; arrows: indicate primer annealing regions; cross bars: indicate the methylated chromosome.

*HUMARA* assay (Figure 1) examines the methylation status of the X chromosome at the *HUMARA* gene; this is a highly polymorphic locus, which allows the distinction between the paternal and maternal alleles that carry a different number of -CAG- repeats [15]. The assay is based on digestion of tumor DNA with the methylation sensitive HpaII restriction enzyme that can only digest the unmethylated allele by recognizing the -CCGG- sequence within the *HUMARA* gene. After digestion, the methylated, therefore ‘protected’ allele, is amplified by PCR; the *HUMARA* -CAG- repeats are approximately 100 bp from the HpaII restriction site, allowing amplification and detection of a portion of the methylated allele, which remains intact after digestion [15]. PCR products are then analyzed by electrophoresis to determine allele number and quantify allele intensity. Using this assay, a monoclonal tumor population expanded from a single cell of origin, carrying the same inactivated chromosome in all cells, will result in a single PCR product, while two PCR products of different molecular size will indicate a polyclonal tumor cell population, where the tumor cells are derived from more than one cell of origin (Figure 1). The extent of allele representation in a tissue is then determined by comparing the amount of PCR products obtained from the two digested alleles to mock-digested samples. This compensates for the preferential amplification of the smaller allele. The allele ratio found in the tumor sample is then divided by the allele ratio measured in DNA from nearby normal tissue, to correct for possible skewed Lyonization, a phenomenon of unbalanced, non-random X chromosome inactivation that occurs in about 10% of a healthy female population [16].

### Laser tissue microdissection and DNA extraction

*HUMARA* clonality assessment [11,15,17] was conducted on stage I to III MM biopsies (Table 1). A limitation of this test is that, it can be used only on female specimens. Although MM is rare in women, we were able to collect and study clonality in 16 MM biopsies from 14 women that were treated by some of the co-authors. In 2 out of 14 cases, two distinct nodules from the same pleura were available for comparison. Tumors and adjacent normal tissues were dissected by Laser Capture Microdissection, using MMI CellCut Plus (Molecular Machines & Industries, MI, USA). Hematoxylin and Eosin (H&E) staining was used to help identify tissue purity. Microdissection was performed on serial sections stained with Hematoxilin (i.e., cut next to the one stained with H/E) on tumor areas containing less than 5% of infiltrating inflammatory cells and in areas containing only normal tissue (control) (Additional file 1: Figure S1).

*HUMARA* assay was performed on DNA extracted from microdissected tissues. DNA was extracted from microdissected tissue using the QiAamp DNA Micro Kit DNA Extraction Protocol (Qiagen). DNAs were then digested with HpaII enzyme as previously described [17]: briefly, 100 ng of either tumor or normal DNA were digested with 10 U Hpa II restriction enzyme (New England Biolabs, Ipswich, MA, USA) at 37°C overnight in a 20 μl reaction volume. Separate aliquots of DNA were subjected to mock digestion without the enzyme. After incubation, the restriction enzyme was inactivated at 80°C for 20 min. HpaII-digested or mock-digested DNA was then subjected to PCR reaction, using the following primers: 5 FAM-labeled forward primer, 5’ACC GAG GAG CTT TCC AGA AT3’; reverse primer, 5’TGG GGA GAA CCA TCC TCA C3’. Thermal cycling conditions included the following steps: denaturation at 95°C for 10 minutes; 30 cycles at 95°C for 30 seconds, 55°C for 30 seconds, and 72°C for 30 seconds; and a final extension at 72°C for 10 minutes. Products of PCR amplification were analyzed by gel and capillary electrophoresis. Gel electrophoresis was performed on 3% agarose gel containing ethidium bromide (10ug/ml), and resolved DNA bands were visualized on a UV transilluminator (Biorad). For capillary electrophoresis, PCR products were mixed with 95% formamide and loading buffer (5% blue dextran, 25 mM EDTA) containing Rox-500. The mixture was then loaded on a 5% Long Ranger–6 M urea gel in TBE buffer. Electrophoresis was performed at 200 W for 2.25 hours, and the data were analyzed by an on a ABI 3100 Genetic Analyzer (Applied Biosystems, Foster City, CA) and quantified by Genescan 3.1 software (Applied Biosystems). Mock-digested samples were used to monitor possible false positive results and to correct allele ratios. All samples were analyzed in triplicate. The ratio SD/mean CR was <2% for all replicates indicating that results were 100% reproducible and reliable.

### Data analysis

For each sample, the allele intensities were measured as the peak areas of both alleles, which are proportional to the molar amount of DNA. The allele ratios (AR) were first calculated by dividing the ratio (R_U_ = A1_U_/A2_U_) of the non-HpaII digested sample by the ratio (R_D_ = A1_D_/A2_D_). The AR calculation (AR = R_U_/R_D_) corrects for any preferential amplification of one allele that might occur if the alleles are different in length. The clonality ratio (CR) is then calculated by dividing the AR of the tumor DNA by the AR calculated for the normal tissue (CR = AR_T_÷AR_N_). This final calculation corrects for a potential skewed Lyonization [18,19]. A CR ≥3.0 or ≤0.33, representing a preferential loss of intensity in the digested sample of one of the two alleles present in the tumor sample, was scored as a monoclonal pattern [18,19].

The Wald method was used to calculate the confidence interval for the observed proportion of polyclonality in our sample population.

## Results and discussion

**Figure 2:**
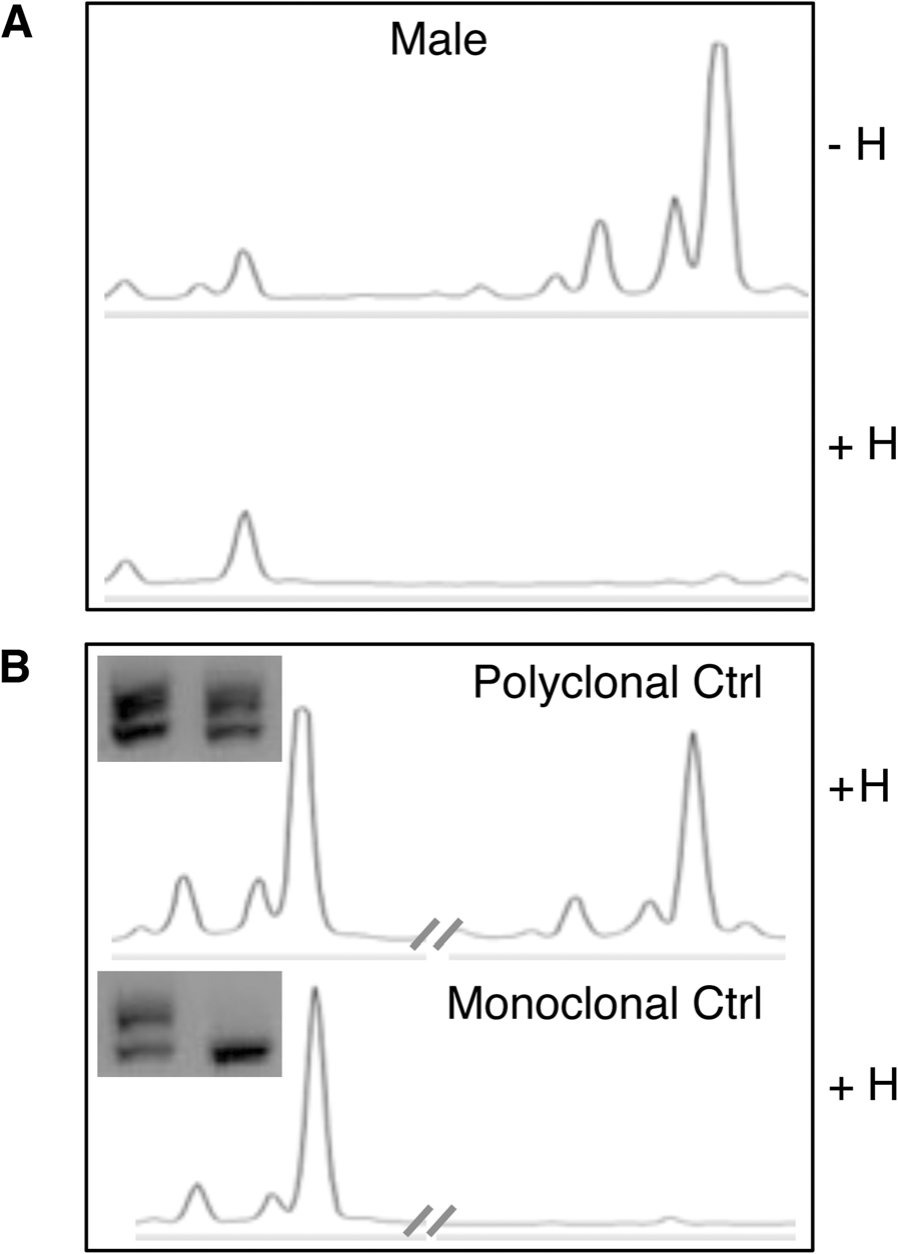
***HUMARA*** **assay quality controls.** *HUMARA*-PCR was performed on HpaII-digested (H+) and mock-digested DNA (H-) from a healthy male and analyzed by capillary gel electrophoresis, using the 3100 Genome Analyzer. Presence of a single PCR peak indicated complete DNA digestion by HpaII enzyme **(A)**. Healthy female DNA sample (L-IV-II, upper panel) and DNA from a human monoclonal melanoma cell line (#1290, female, lower panel) were subjected to *HUMARA* assay. After HpaII digestion, gel (inserts) and capillary electrophoresis successfully detected a polyclonal pattern and a monoclonal pattern, respectively **(B)**.

To address whether MM have monoclonal or polyclonal origin, we performed the *HUMARA* assay, as described in Methods and Figure 1 [11,15,17]. We first performed the *HUMARA* assay on control samples (Figure 2). A healthy male DNA sample (monoallelic, bearing a single unmethylated X chromosome) was digested as described in the material and methods and analyzed by agarose gel and capillary electrophoresis. Disappearance of the PCR band/peak indicated complete allele digestion, thus excluding a bias in our *HUMARA* assay due to incomplete HpaII digestion (Figure 2A). A healthy female DNA sample (L-IV-II, biallelic) and a known monoclonal melanoma cell line (#1290, female) were used as additional controls. As shown in Figure 2B, gel and capillary electrophoresis successfully detected a polyclonal and a monoclonal pattern, respectively.

**Figure 3:**
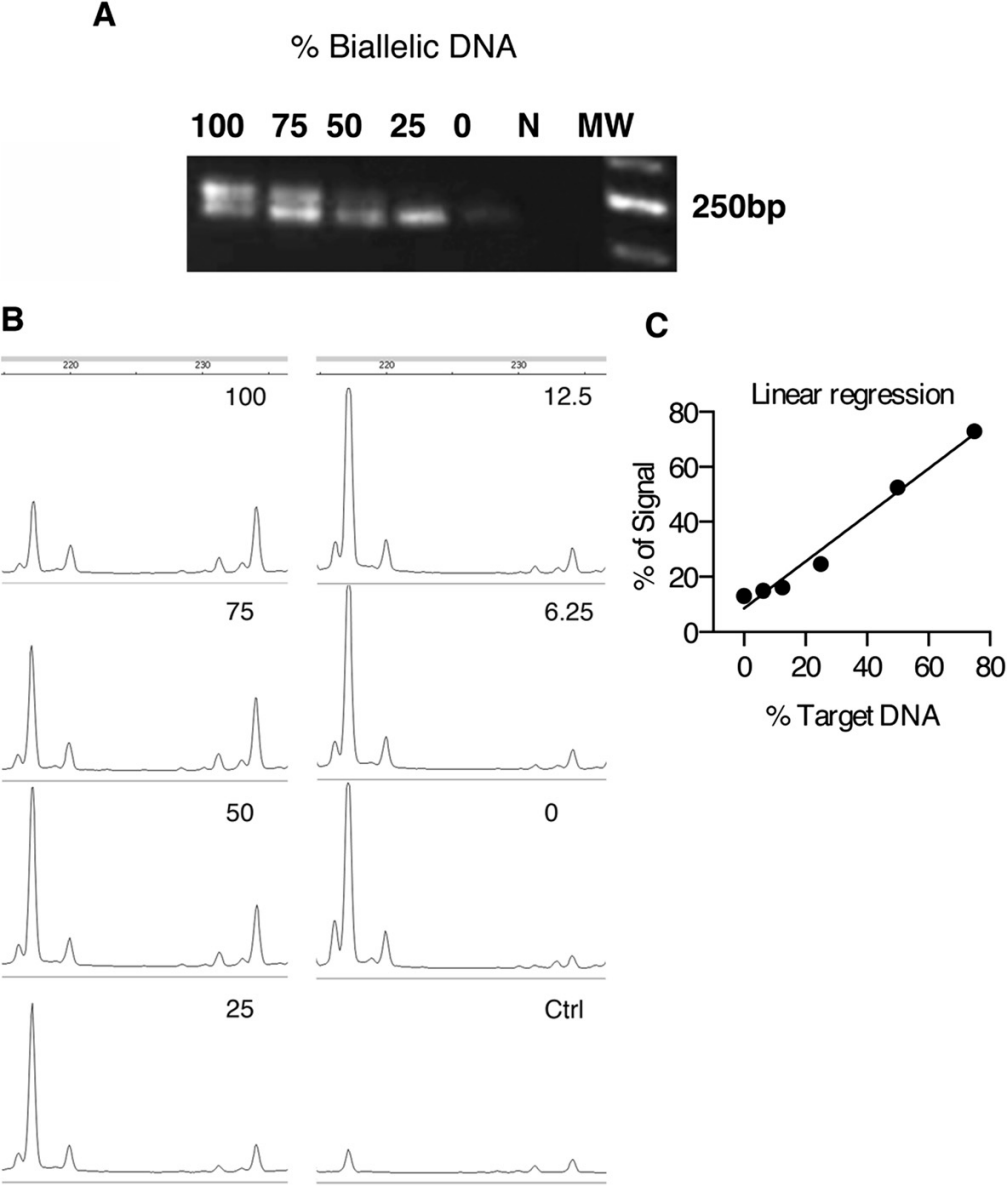
**Sensitivity of the** ***HUMARA*** **assay by gel and capillary electrophoresis.** To establish the sensitivity of the *HUMARA* assay for detection of minor alleles, different amounts of HpaII-digested (mono-allelic) and non-digested (representing bi-allelic DNA) #1290 DNA were mixed in different rations and subjected to PCR for detection of *HUMARA* locus. PCR products were resolved on a 3% agarose gel containing 0.5 ug/ml ethidium bromide and detected under UV light **(A)** or using the Applied Biosystems 3100 Genetic Analyzer **(B)**. CTR denotes the no template control. 100, 75, 50, 25, 12.5, 6.25 and 0 indicate the percentage of bi-allelic DNA in the PCR reaction **(A)**. Linear regression analysis calculated with Prism 6 software, shows comparison between input and detected allelic/biallelic ratios as calculated using Genescan software. (R^2^ > 0.98). The minor allele was reliably detectable when present at a fraction greater than or equal to 1/8 (12.5% of the input copies) **(C)**.

Next, we established the sensitivity of the *HUMARA* assay in the detection of under-represented alleles by both gel and capillary electrophoresis (Figure 3). Mono-allelic (HpaII-digested) and bi-allelic (non-digested) DNA from the 1290 melanoma monoclonal cell line were mixed in different proportions. PCR products were resolved by electrophoresis on 3% agarose gel and visualized under UV light in the presence of ethidium bromide (Figure 3A), or by capillary electrophoresis using a Genotypic Bioanalyzer (Applied Biosystems) (Figure 3B). Linear regression of input vs detected allele ratios revealed a robust correlation (R^2^ > 0.98), with the less frequent allele detectable when present at a fraction greater than or equal to 1/8 (12.5% of the input copies) (Figure 3C).

**Figure 4:**
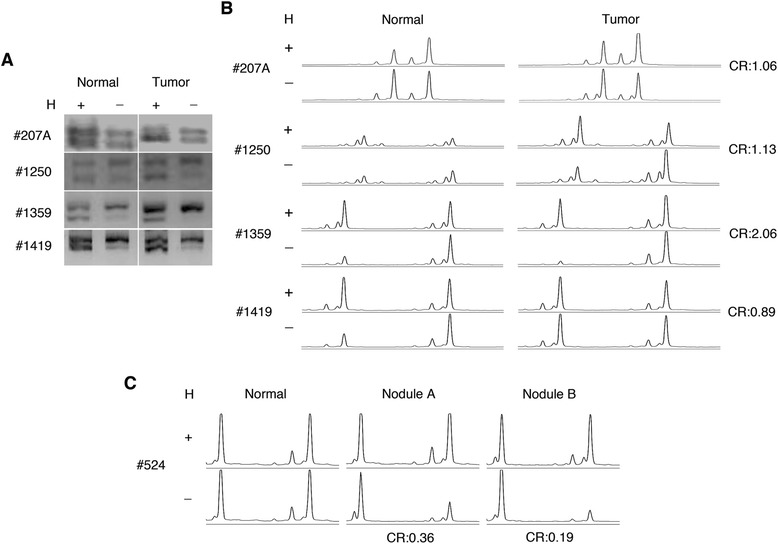
**X chromosome inactivation analysis by** ***HUMARA*** **assay shows a prevalent polyclonal pattern of Malignant Mesotheliomas.** Gel electrophoresis (A). PCR products from mock digested (H-) and HpaII-digested (H+) DNA samples were separated on a 3% agarose gel and visualized under UV light, using ethidium bromide. Capillary-Electrophoresis **(B, C)**.* HUMARA* PCR assay was performed using a 5FAM-labeled forward primer, and quantified by the Applied Biosystems 3100 Genetic Analyzer. Two major peaks denote the two allelic *HUMARA* loci PCR-amplified in HpaII-digested (H+) and mock-digested samples (H-). The allele intensities were measured as peak area of both alleles, which is proportional to the molar amount of DNA. Peak areas were calculated for each allele using Genescan software, as described in the Material and Methods. A CR ≥3.0 or ≤0.33, representing a preferential loss of intensity in the digested sample of one of the two alleles present in the tumor sample, was scored as a monoclonal pattern.

*HUMARA* clonality assessment was conducted on 16, stage I to III, frozen biopsies from 14 cases of female MMs (Table 1). In 2 out of 14 sporadic cases, two distinct nodules from the same pleura were available for analysis, giving a total of 16 specimens. Samples were classified as non-informative when a single band or peak was detected in the nearby normal tissue after digestion with the HpaII enzyme, indicating the presence of skewed Lyonization. Out of the 16 biopsies tested, 15 were informative. Sample L-III-18, a case of familial MM, was non-informative as PCR amplification of both mock- and HpaII-digested DNA produced a single band, suggesting that the lengths of the paternal and maternal alleles were identical (data not shown). Of the 15 informative biopsies, 14/15 PCR products (93.3%) displayed two distinct bands and peaks. 4 representative samples are shown in Figure 4A and B. Corrected Allele Ratio (CR) calculated on the allele peak areas by Genotypic Bioanalyzer was within the values of 0.33 and 3.0, indicating polyclonality of all 14 samples (Figure 4B and Table 1). The observed proportion of polyclonality was 93.33% (14/15), with a 95% confidence interval of [68.16% - 100%], indicating that the proportion of polyclonality in our sample population did not differ significantly from 100%. Therefore our results reveal with confidence that MM are polyclonal at origin. Case #524 showed a quite distinct pattern, as one nodule (#524B) revealed a monoclonal pattern (CR = 0.19), while the other (#524A) was polyclonal (CR = 0.36; Figure 4C). This finding suggested that a particular clone may dominate a certain area within a largely polyclonal tumor, composed of clones derived from different cells of origin. Of note, both sporadic MMs, that usually develop on a background of asbestos exposure, and familial MMs that develop on a background of germline *BAP1* mutations were found to be polyclonal. All these MM samples were from women, as the *HUMARA* assay used here to assess clonality, cannot be used on specimen from men. MMs in women are rare and have a slightly better prognosis than MM in man [20], a finding that some studies suggested might be related to the expression of the estrogen receptor beta [21]. However, there is no evidence in the literature supporting the hypothesis that women and man MM have a different pathogenesis, thus it seems unlikely that polyclonality of MM is influenced by gender-associated factors. It should also be noted that all these MMs were either of the epithelial or of the biphasic type (Table 1), which account for 90% of MMs. We did not test sarcomatoid MMs that account for about 10% of all MMs, because these specimens were not available, as they are rare and as these patients are not candidates for surgical resection [2]. Thus, clonality of sarcomatoid MMs remains to be tested.

**Figure 5:**
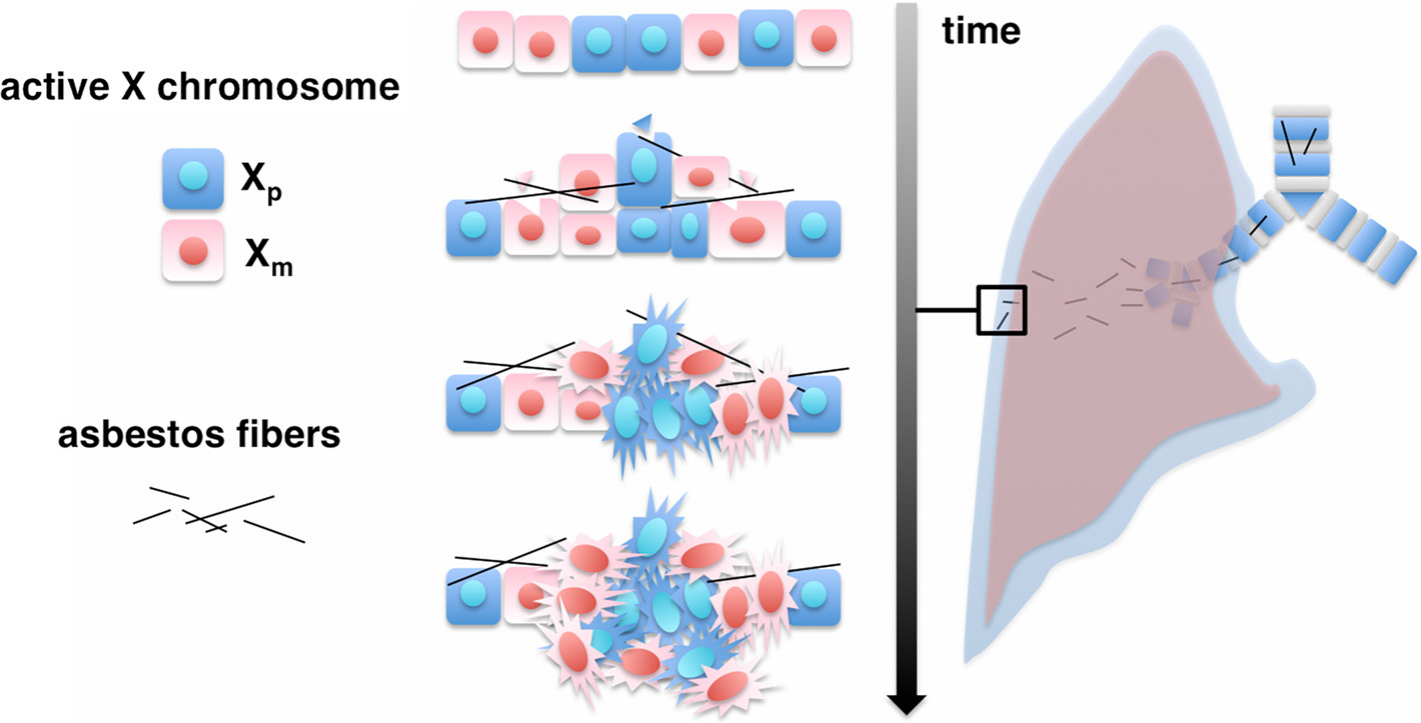
**MMs originate as polyclonal tumors.** Asbestos fibers travel through the airways to the lungs, and, from there, via the lymphatics, reach the pleura, exposing many mesothelial cells. The carcinogenic “field effect” of mineral fibers leads to several premalignant clones that give rise to these polyclonal malignancies.

X-chromosome inactivation based assays may detect a seemingly monoclonal tumor when transformation occurs in multiple cells having the same inactivated X chromosome. Although possible, this is a rare event, mainly dependent on the X-inactivation patch size during early developmental Lyonization. This event leads few of the progeny of a single embryonic stem cell to be grouped together in the adult, forming patches. This phenomenon has been described in breast tissues, which display a rather large patch size [12]. Alternatively, a true monoclonal nodule may result from the clonal outgrow of a particular cell with proliferative advantage, as previously described in breast and prostate cancer, e.g. due to acquisition of LOH of the X-linked tumor suppressor *FOXP3* [22,23]. The notion that tumors derive from a single cell through the expansion and evolution of several clones has survived mostly unchallenged until the present time. Accordingly, much effort has been placed in using genomic approaches to dissect the clonal relationships present within single tumors [8]. Despite the advances of the molecular genetic analyses, to date, the *HUMARA* assay is unique in its capability of detecting the actual origin of a cell population. Our data indicate that MMs may arise as polyclonal tumors due to concurrent transformation of multiple mesothelial cells (Figure 5). Recent studies conducted in some breast and colon carcinomas showed that they were polyclonal [13,14,24,25], supporting the novel concept that not all tumors are monoclonal at origin. Also, a recent study using mouse chimeras, provides evidences suggesting that intestinal tumors may be polyclonal and that inter-clonal interactions are necessary for tumor development [13]. Our results that MMs are polyclonal indicate that MMs are likely to be clonally complex at the outset. The polyclonal origin of MM may account for the very high degree of intra-tumoral heterogeneity found in these malignancies, and also contribute to the emergence of drug-resistant subpopulations. Therefore, our finding suggest that solely tracking the clonal evolution of a predominant clone may not be sufficient to successfully target MM, providing a possible rationale to the peculiar resistance of MM to current therapies.

## Conclusions

Our data indicate that MM arise as polyclonal tumors, a finding that has both pathogenesis and clinical implications. For example, MM patients whose tumors are removed at Stage Ia, most often experience recurrence after surgery in spite of apparent successful MM eradication [20].

Our data suggest that, in contrast to current dogma, recurrence may represent novel malignancies, occurring because of the carcinogenic “field effect” of asbestos, its related chronic inflammation, and/or because of ubiquitous genetic predisposition in patients carrying germline *BAP1* mutations. Therefore, MM may arise from a large pool of independent and mostly covert cancers, as observed in some other malignancies [26]. Accordingly, the multiple minuscule pleural nodules that are characteristically found on the pleura of early-stage MM patients are likely pre-malignant lesions or early tumors rather than early local metastases. Our findings underscore the need to attack simultaneously several different molecular targets to try to eliminate the different MM cell clones, as each clone may carry its own distinct set of molecular alterations.

## Additional file

Additional file 1: Figure S1. Laser capture microdissection of MM. H&E of a representative MM tumor section is shown before **(A)** and after **(B)** tumor tissue collection by laser capture microdissection (200x magnification).

## Abbreviations

MM: Malignant Mesothelioma
*HUMARA*: Human androgen receptor
H&E: Hematoxylin and Eosin
AR: Allele ratio
CR: Corrected ratio

## References

[1] Henley SJ, Larson TC, Wu M, Antao VC, Lewis M, Pinheiro GA, Eheman C (2013). Mesothelioma incidence in 50 states and the District of Columbia, United States, 2003-2008. Int J Occup Environ Health.

[2] Carbone M, Ly BH, Dodson RF, Pagano I, Morris PT, Dogan UA, Gazdar AF, Pass HI, Yang H (2012). Malignant mesothelioma: facts, myths, and hypotheses. J Cell Physiol.

[3] Carbone M, Baris YI, Bertino P, Brass B, Comertpay S, Dogan AU, Gaudino G, Jube S, Kanodia S, Partridge CR, Pass HI, Rivera ZS, Steele I, Tuncer M, Way S, Yang H, Miller A (2011). Erionite exposure in North Dakota and Turkish villages with mesothelioma. Proc Natl Acad Sci U S A.

[4] Baumann F, Ambrosi JP, Carbone M (2013). Asbestos is not just asbestos: an unrecognised health hazard. Lancet Oncol.

[5] Carbone M, Yang H (2012). Molecular pathways: targeting mechanisms of asbestos and erionite carcinogenesis in mesothelioma. Clin Cancer Res.

[6] Carbone M, Yang H, Pass HI, Krausz T, Testa JR, Gaudino G (2013). BAP1 and cancer. Nat Rev Cancer.

[7] Husain AN, Colby T, Ordonez N, Krausz T, Attanoos R, Beasley MB, Borczuk AC, Butnor K, Cagle PT, Chirieac LR, Churg A, Dacic S, Fraire A, Galateau-Salle F, Gibbs A, Gown A, Hammar S, Litzky L, Marchevsky AM, Nicholson AG, Roggli V, Travis WD, Wick M, International Mesothelioma Interest G (2013). Guidelines for pathologic diagnosis of malignant mesothelioma: 2012 update of the consensus statement from the International Mesothelioma Interest Group. Arch Pathol Lab Med.

[8] Aparicio S, Caldas C (2013). The implications of clonal genome evolution for cancer medicine. N Engl J Med.

[9] Greaves M, Maley CC (2012). Clonal evolution in cancer. Nature.

[10] Shibata D (2012). Cancer. Heterogeneity and tumor history. Science.

[11] Chen GL, Prchal JT (2007). X-linked clonality testing: interpretation and limitations. Blood.

[12] Novelli M, Cossu A, Oukrif D, Quaglia A, Lakhani S, Poulsom R, Sasieni P, Carta P, Contini M, Pasca A, Palmieri G, Bodmer W, Tanda F, Wright N (2003). X-inactivation patch size in human female tissue confounds the assessment of tumor clonality. Proc Natl Acad Sci U S A.

[13] Thliveris AT, Schwefel B, Clipson L, Plesh L, Zahm CD, Leystra AA, Washington MK, Sullivan R, Deming DA, Newton MA, Halberg RB (2013). Transformation of epithelial cells through recruitment leads to polyclonal intestinal tumors. Proc Natl Acad Sci U S A.

[14] Parsons BL (2008). Many different tumor types have polyclonal tumor origin: evidence and implications. Mutat Res.

[15] Allen RC, Zoghbi HY, Moseley AB, Rosenblatt HM, Belmont JW (1992). Methylation of HpaII and HhaI sites near the polymorphic CAG repeat in the human androgen-receptor gene correlates with X chromosome inactivation. Am J Hum Genet.

[16] Sharp A, Robinson D, Jacobs P (2000). Age- and tissue-specific variation of X chromosome inactivation ratios in normal women. Hum Genet.

[17] Shattuck TM, Westra WH, Ladenson PW, Arnold A (2005). Independent clonal origins of distinct tumor foci in multifocal papillary thyroid carcinoma. N Engl J Med.

[18] Kopp P, Jaggi R, Tobler A, Borisch B, Oestreicher M, Sabacan L, Jameson JL, Fey MF (1997). Clonal X-inactivation analysis of human tumours using the human androgen receptor gene (HUMARA) polymorphism: a non-radioactive and semiquantitative strategy applicable to fresh and archival tissue. Mol Cell Probes.

[19] Willman CL, Busque L, Griffith BB, Favara BE, McClain KL, Duncan MH, Gilliland DG (1994). Langerhans'-cell histiocytosis (histiocytosis X)–a clonal proliferative disease. N Engl J Med.

[20] Flores RM, Riedel E, Donington JS, Alago W, Ihekweazu U, Krug L, Rosenzweig K, Adusumilli PS, Carbone M, Pass HI (2010). Frequency of use and predictors of cancer-directed surgery in the management of malignant pleural mesothelioma in a community-based (Surveillance, Epidemiology, and End Results [SEER]) population. J Thorac Oncol.

[21] Pinton G, Brunelli E, Murer B, Puntoni R, Puntoni M, Fennell DA, Gaudino G, Mutti L, Moro L (2009). Estrogen receptor-beta affects the prognosis of human malignant mesothelioma. Cancer Res.

[22] Wang L, Liu R, Li W, Chen C, Katoh H, Chen GY, McNally B, Lin L, Zhou P, Zuo T, Cooney KA, Liu Y, Zheng P (2009). Somatic single hits inactivate the X-linked tumor suppressor FOXP3 in the prostate. Cancer Cell.

[23] Zuo T, Wang L, Morrison C, Chang X, Zhang H, Li W, Liu Y, Wang Y, Liu X, Chan MW, Liu JQ, Love R, Liu CG, Godfrey V, Shen R, Huang TH, Yang T, Park BK, Wang CY, Zheng P, Liu Y (2007). FOXP3 is an X-linked breast cancer suppressor gene and an important repressor of the HER-2/ErbB2 oncogene. Cell.

[24] Xin L (2013). Cells of origin for cancer: an updated view from prostate cancer. Oncogene.

[25] Parsons BL (2011). Monoclonal tumor origin is an underlying misconception of the RESIC approach. Proc Natl Acad Sci U S A.

[26] Potter NE, Greaves M (2014). Cancer: Persistence of leukaemic ancestors. Nature.

