# Supplementary material for: Evaluation of clonal origin of malignant mesothelioma
Source: J Transl Med. 2014 Dec 4;12:301. doi: 10.1186/s12967-014-0301-3 (PMC4255423; doi:10.1186/s12967-014-0301-3)
Supplement: Additional file 1: Figure S1. — Laser capture microdissection of MM. H&E of a representative MM tumor section is shown before (A) and after (B) tumor tissue collection by laser capture microdissection (200x magnification). [file 12967_2014_301_MOESM1_ESM.docx]

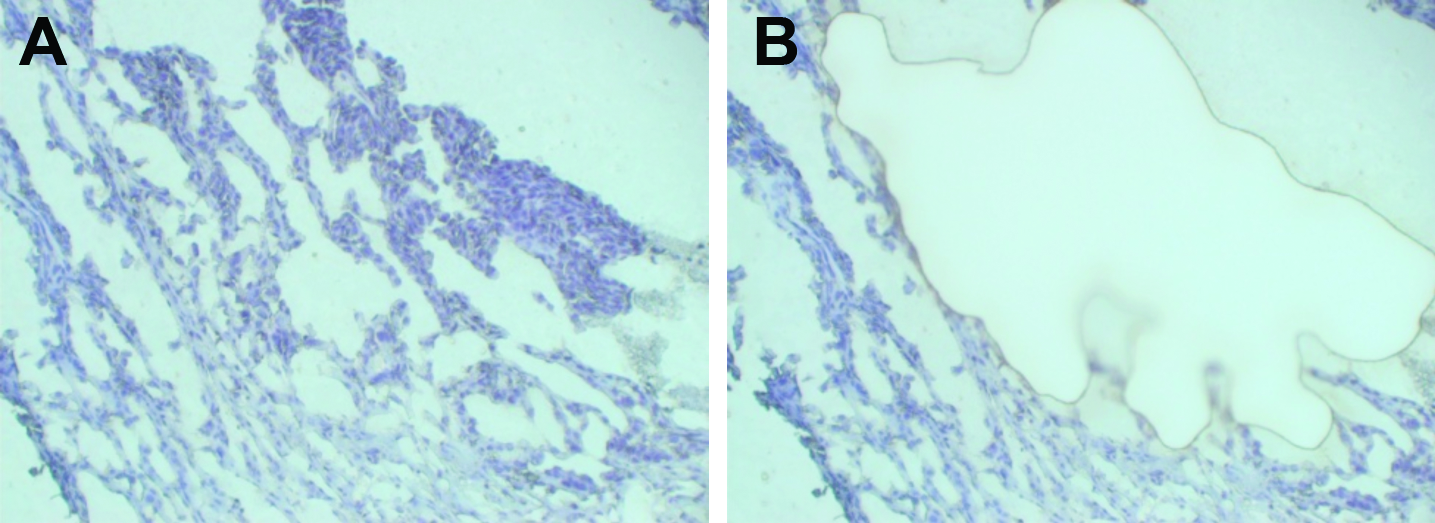


**Additional file 1: Figure S1. Laser capture microdissection of MM.**H&E of a representative MM tumor section is shown before **(A)** and after **(B_­­­_)** tumor tissue collection by laser capture microdissection (200x magnification).
